# isomiRdb: microRNA expression at isoform resolution

**DOI:** 10.1093/nar/gkac884

**Published:** 2022-10-16

**Authors:** Ernesto Aparicio-Puerta, Pascal Hirsch, Georges P Schmartz, Tobias Fehlmann, Verena Keller, Annika Engel, Fabian Kern, Michael Hackenberg, Andreas Keller

**Affiliations:** Clinical Bioinformatics, Saarland University, 66123 Saarbrücken, Germany; Clinical Bioinformatics, Saarland University, 66123 Saarbrücken, Germany; Clinical Bioinformatics, Saarland University, 66123 Saarbrücken, Germany; Clinical Bioinformatics, Saarland University, 66123 Saarbrücken, Germany; Rejuvenome, Astera Institute, Berkeley, CA 94705, USA; Clinical Bioinformatics, Saarland University, 66123 Saarbrücken, Germany; Department for Internal Medicine II, Saarland University Hospital, 66421 Homburg, Germany; Clinical Bioinformatics, Saarland University, 66123 Saarbrücken, Germany; Clinical Bioinformatics, Saarland University, 66123 Saarbrücken, Germany; Helmholtz Institute for Pharmaceutical Research Saarland (HIPS)–Helmholtz Centre for Infection Research (HZI), Saarland University Campus, 66123 Saarbrücken, Germany; Genetics Department, Faculty of Science, Universidad de Granada, 18071 Granada, Spain; Clinical Bioinformatics, Saarland University, 66123 Saarbrücken, Germany; Helmholtz Institute for Pharmaceutical Research Saarland (HIPS)–Helmholtz Centre for Infection Research (HZI), Saarland University Campus, 66123 Saarbrücken, Germany

## Abstract

A significant fraction of mature miRNA transcripts carries sequence and/or length variations, termed isomiRs. IsomiRs are differentially abundant in cell types, tissues, body fluids or patients’ samples. Not surprisingly, multiple studies describe a physiological and pathophysiological role. Despite their importance, systematically collected and annotated isomiR information available in databases remains limited. We thus developed isomiRdb, a comprehensive resource that compiles miRNA expression data at isomiR resolution from various sources. We processed 42 499 human miRNA-seq datasets (5.9 × 10^11^ sequencing reads) and consistently analyzed them using miRMaster and sRNAbench. Our database provides online access to the 90 483 most abundant isomiRs (>1 RPM in at least 1% of the samples) from 52 tissues and 188 cell types. Additionally, the full set of over 3 million detected isomiRs is available for download. Our resource can be queried at the sample, miRNA or isomiR level so users can quickly answer common questions about the presence/absence of a particular miRNA/isomiR in tissues of interest. Further, the database facilitates to identify whether a potentially interesting new isoform has been detected before and its frequency. In addition to expression tables, isomiRdb can generate multiple interactive visualisations including violin plots and heatmaps. isomiRdb is free to use and publicly available at: https://www.ccb.uni-saarland.de/isomirdb.

## INTRODUCTION

MicroRNAs (miRNAs) are small non-coding RNA molecules that mediate gene silencing by promoting translation repression and degradation of target mRNAs (1), mostly by binding to reverse complementary sites in 3′ UTR regions (2). At least 60% of mRNAs are miRNA targets (3) so it's not surprising that their dysregulation is frequently associated with pathologies (4). Early sequencing data revealed that, in their mature form, most miRNAs naturally display sequence and length variations (5) that can arise from imprecise Drosha and Dicer cleavage or non-templated nucleotide additions (6). Said isoforms, commonly termed isomiRs, can affect miRNA targeting (7) and stability and are differentially abundant in developmental stages (7), cell types (8), tissues, bodily fluids (9) or samples from patients, including cancer (10). Additionally, several studies have described important physiological roles of isomiRs based on altered targeting compared to their canonical counterparts (11) as well as biomarker potential for disease detection, diagnosis and prognosis (10).

Despite their apparent importance, systematically collected and annotated isomiR information available in resources remains limited. Of note, a substantial number of miRNA databases exists including those that collect sequence information (12), disease relation (13) and expression across organisms (14), tissues (15) and cell-types (16). Many of these databases compile miRNA-seq datasets from a wide spectrum of origins but only a couple provide isomiR expression information (Table 1). Furthermore, although publicly available miRNA-seq samples are rapidly increasing (17), several current resources compile expression data for a limited number of samples. Here, DIANA-miTED (18) is a remarkable exception hosting over 15 000 datasets. Among the available solutions, only IsomiR Bank (19) and Tumor IsomiR Encyclopedia (TIE) (20) include isomiR expression data. IsomiR Bank from 2016 excels by covering expression data for eight species and is based on 2 727 samples. TIE is an agile recent resource to explore human isomiRs from over 10 000 samples. It stands out by the clear focus on cancer, relying on TCGA data. Given the importance of isomiRs, a comprehensive resource compiling extensive miRNA profiles at isoform resolution seems to be of high value for the miRNA research community.

**Table 1.** MiRNA and isomiR expression resources. All of them are accessible as online databases except the human cellular microRNAome, which is both an R package and a track at UCSC Genome Browser

Resource	Number of miRNA-seq datasets	Number of organisms	Summary of features	IsomiR data	Last release date
Deepbase	21 235	1 (Human)	Integration of different ncRNA profiles	No	2021
SEAweb	>42 00	10	Also provides pathogen information	No	2020
microRNAome package	2 077	1 (Human)	Manually curated cell types	No	2022
miRNATissueAtlas	188	2 (Human and mouse)	188 tissue samples from 6 individuals	No	2022
DIANA-miTED	15 183	1 (Human)	Manually curated tissues	No	2022
IsomiR Bank	2 727	8	IsomiR target prediction and enrichment analysis	Yes	2016
Tumor IsomiR Encyclopedia	11 667	1 (Human)	IsomiRs from TCGA	Yes	2021
isomiRdb	42 499	1 (Human)	Largest compilation of isomiRs	Yes	2022

**Figure 1.**
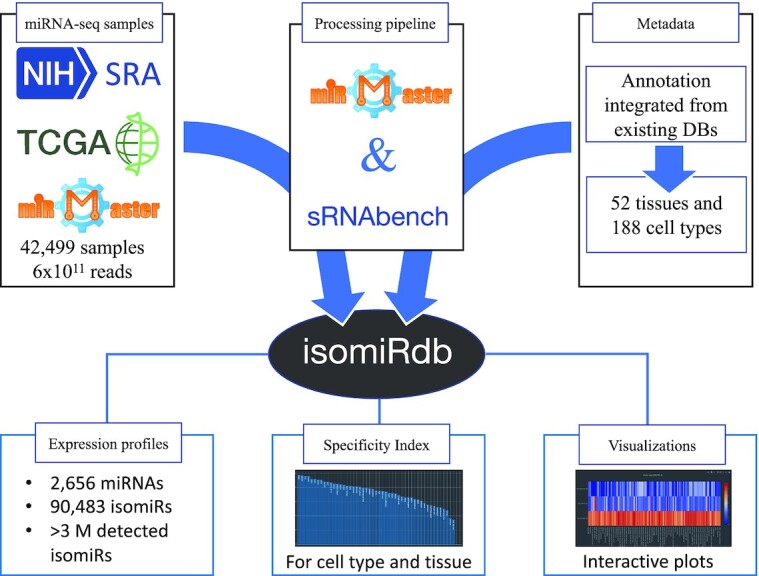
Development process and functionalities of isomiRdb. In short, 42 499 miRNA-seq samples from TCGA, SRA and provided by miRMaster users were uniformly processed to offer tissue and cell type expression levels of miRNAs at isomiR resolution. Tissue/cell specificity index and several types of visualizations are available.

Here, we present isomiRdb, a miRNA expression database at isoform level compiling over 90 483 isomiRs (Figure 1). The resource also provides relevant information for another ∼3 million lower abundance isoforms (all called isomiRs down to a single sample). The database builds on samples from the Sequence Read Archive (SRA) (21), the Cancer Genome Atlas (TCGA) and profiles analyzed by miRMaster (22) reaching a total of 42 499 miRNA-seq datasets. Together, these small RNA sequencing data compile 5.9 × 10^11^ processed reads, making it a very comprehensive and powerful miRNA expression resource. To present comparable data, we analyzed all data sets from raw fastq files that were uniformly pre-processed with miRMaster followed by downstream processing by sRNAbench (17). The annotation of miRNAs relies on the latest version of miRbase (release 22.1) (12). As described in detail below, we curated isomiRs using different quality control flags depending on the reported activity of enzymes involved in miRNA metabolism to certify the likely biological nature of said isoforms or warn about likely artifacts. Furthermore, we combined metadata from different sources to annotate isomiRdb samples with 52 tissues and 188 cell types. In sum, the database excels by the large number of samples, extensive number of sequencing reads and its detailed annotation. Combining these strengths, we provide a unique resource hosting miRNA expression data and isoform information at isomiR resolution. Access to the data is possible in multiple ways. Users can query expression values by sample, miRNA or isomiR and several interactive visualizations are available as well as tabular files ready for download. In addition to the 90 483 higher abundance isomiRs included in the online expression repository, we decided to release a list of all detected isomiRs. This set of over 3 million potential isomiRs is available on the database for query and download. isomiRdb is free to use and publicly available at: https://www.ccb.uni-saarland.de/isomirdb.

## DATA AND METHODS

### Generation of miRNA and isomiR profiles

We compiled 42 499 miRNA-seq human datasets from three different sources: SRA, TCGA and samples that were made accessible by miRMaster users who provided consent for aggregated secondary usage of their data, as similarly done before (23). We identified duplicated samples (i.e. SRA samples that were also uploaded to miRMaster) and removed them to avoid bias. To further minimize technical variations, we started all analyses from the raw fastq files. Here, we combined the advantages of different computational tools. First, the raw read files were pre-processed with miRMaster (22) to detect library preparation protocol, trim and collapse reads using default parameters. MiRMaster performs adapter trimming via fuzzy string matching and read quality control applying a sliding window approach (22). Subsequently, collapsed reads were profiled with sRNAbench (17) in library mode and default parameters using miRBase release 22.1 (12). Essentially, sRNAbench relies on bowtie (24) for read alignment (with parameters *-f -n 1 -l 19 -a –best –strata*) and multiple-mapping reads are evenly split among receiving sites.

MiRNA and isomiR expression data were stored in the database considering different units: read counts (RC), reads per million (RPM, normalized to miRNA reads) and fraction of parent miRNA reads assigned to the isomiR. For miRNAs with several loci, we considered the average value across all loci. In total, isomiRdb offers miRNA expression values for all 2 656 mature miRNAs hosted in miRBase and for the 90 483 most abundant isoforms (>1 RPM in at least 1% of the samples). A list of over 3 million isoforms with lower abundance but still detected in at least one sample is available for download.

### isomiR classification system

isomiRdb relies on sRNAbench's hierarchical isomiR classification system (25). Except for canonical isoforms (i.e. the exact sequence present in the reference database), all other isomiRs are matched to one or several of the following four classes. (A) Nucleotide Variant (NucVar): nucleotide variations in the canonical sequence. (B) 5′ length variants (lv5p): 5′ end is shorter or longer but coincidental with the template sequence. (C) 3′ length variants (lv3p): 3′ end is shorter or longer but coincidental with the template sequence. (D) Non-templated additions (NTA): enzymatic post-transcriptional addition that is not included in the template sequence (e.g. NTA#T denotes an uridylation). Finally, isomiRs belonging to several classes are labelled as multiple variants (mv).

### Quality control of putative miRNA isoforms

To provide users with improved confidence on isoforms that are the likely result of miRNA metabolism or to discard potential artifacts we curated all isomiRs using previously available information. As a result of this quality control, 4 tags have been incorporated: New England's Biolabs (NEB) NTA#C (warning), *bona fide* NTA#U, *bona fide* alternative processing by Drosha and unexpected alternative processing by Drosha (warning). Canonical sequences minus 1 nt at the 3′ end followed by a C or NTA#U followed by a C were flagged as likely artifacts from the NEB protocol. 5′ and 3′ length variants were tagged as *bona fide* or likely artifact after checking if their Drosha processing pattern was consistent with previous reports (26). In short, 5′ length variants of *5p* miRNA arms were labelled as *bona fide* if alternative processing for that miRNA had been previously established by Kim *et al.* (26) or as possible artifacts if they had been classified into the single-site processing group (>95% of cuts on the same site). The equivalent assignation was performed on 3′ length variants of *3p* arms. Finally, we reanalyzed data from a set of TUT7 and TUT4 knockdown experiments (27) to determine which NTA#U isoforms could be the result of enzymatic metabolism and label them accordingly.

### Metadata acquisition

To provide useful results, detailed annotation with metadata is a key step. We thus integrated per sample annotation from several sources to include tissue and cell type information. Tissue annotations are available from previously curated resources including liqDB (28), mirnaQC (29), miRSwitch (23), TCGA and from SRA using OmicIDX. Cell type annotation was obtained from the human cellular microRNAome project (16). In total, 52 different tissues and 188 cell types were annotated as metadata.

### Webserver and database implementation

To provide an efficient and appealing web interface for isomiRdb, we used the Django Web Framework (v.2.2), Bootstrap (v5.0.1), javascript and Plotly (v2.12.1) for interactive visualisations. Sparse representation matrix files (.h5ad) are central for the backend database and loaded with the *anndata* python package (v 0.8.0). To estimate the specificity of tissues, we computed the tissue specificity index (TSI) of each miRNA as previously described (15,30). The same formula was also applied to mean expression of cell types to provide Cell Specificity Index (CSI).

## RESULTS

### Website sections and types of queries

The main functionality of isomiRdb allows querying of expression data at three different levels: samples, miRNAs and isomiRs. We implemented specific subpages for each of the three to account for different analysis aspects. In the *Samples* view, users can retrieve expression distributions of the top 3, 5, 10, 20 or 50 most expressed miRNAs in any tissue or cell-type contained in isomiRdb. MiRNA expression values can be retrieved in three different units: RC, RPM or log_10_(RPM + 1). Expression levels are visualised using well-known concepts such as violin plots, boxplots or bar charts. Of course, the displayed data are available for download in tabular format. Conversely, users can also query miRNAs of interest using the *miRNAs* view. In this case, after choosing a miRNA of interest, isomiRdb provides similar graphical information as in the previous view but with samples grouped by tissue or cell-type. Similarly, we implemented the core view, specific for the *isomiR* information. Here, we provide an interactive table with the option to apply several filters. This table also grants quality information about each isoform by means of tags that can indicate possible artifacts (certain NTA cytosines or unexpected Drosha cuts) or likely *bona fide* isoforms (miRNAs with previously reported biological monouridylation or alternative Drosha processing). Adjusting the sequence, parent miRNA or isomiR category allows to identify any isomiR of interest. After selecting an isomiR, the web server generates and displays all corresponding graphical representations.

As a further option, the resource offers the possibility to explore Tissue Specificity Index and Cell Specificity Index, an established estimate of expression specificity (15,30). In summary, this index provides a standardized measure of how tissue/cell-specific a miRNA is. A value of 1 means the miRNA is specific and only expressed in a particular tissue or cell type while a value of 0 means it is present in every tissue/cell type. Additionally, we provide a tool to interactively visualize heatmaps annotated by miRNA/isomiR and tissue/cell-type. Finally, a *Downloads* section allows to batch download expression data and metadata hosted in isomiRdb. Of note, the resource provides expression values for isomiRs that meet the minimum expression threshold of at least 1 RPM in at least 1% of the samples for two reasons. Showing all isoforms would not only lead to performance restrictions but might also introduce noise to the data. More experienced scientists that are interested in detailed and holistic analyses of the isomiR information can download the lower abundance isomiR repertoire from the web page.

### Use case: ubiquitously expressed miR-21 and its variants

**Figure 2.**
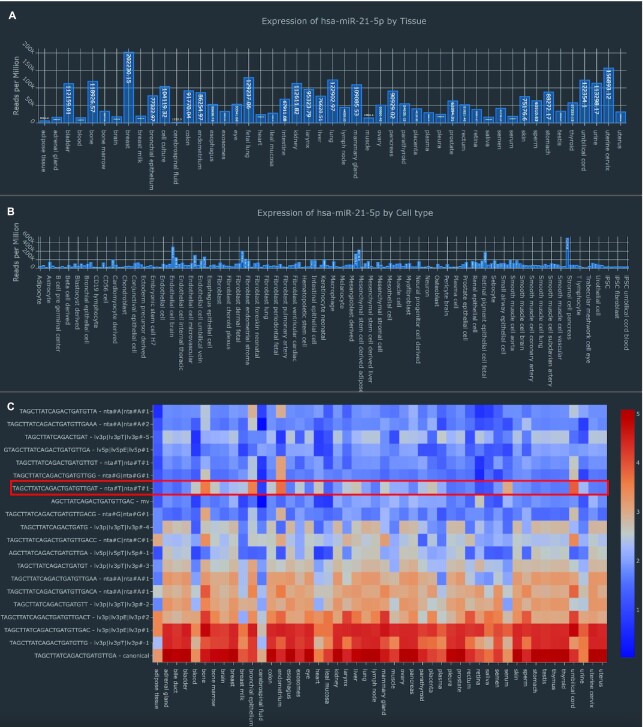
Different visualizations of miR-21–5p expression. (**A**) Mean expression of miR-21-5p per tissue. (**B**) Mean expression of miR-21-5p per cell type. (**C**) Heatmap of miR-21-5p isomiRs. A NTA-U isoform described in the use case is highlighted in red.

There are various application scenarios for our database. It allows for very specific considerations. For example, researchers can make use of the resource to find out whether one isomiR is expressed at all and in which tissues. The evaluation of the complete repertoire of isomiRs for one miRNA is feasible too. Finally, complex analyses are also possible, for example users can explore the isomiR repertoire of one cell type or tissue. As use case we provide insights in miR-21-5p. This miRNA is ubiquitously expressed and has been proposed as a biomarker for at least 29 diseases (31). Using the *miRNAs* view, we can easily check that it is indeed present in every tissue and cell type. In fact, its average expression exceeds 1000 RPM in any cell type or tissue (Figure 2A-B). We next checked the miR-21-5p isoform expression pattern by means of the *Heatmaps* view (Figure 2C). On inspection of the isomiR profile, some variation exists on the most abundant isoforms ranging from canonical sequence to 3′ length variants of ± 1 nt but no general trends are obvious. One isomiR though, *TAGCTTATCAGACTGATGTTGAT*, an NTA-U, exhibits an interesting expression pattern: while in most tissues its expression is between 0–50 RPM, a few tissues have a considerably higher expression with some exceeding 300 RPM (Figure 2C). These tissues include bone, bone marrow, heart, mammary gland, placenta, liver, bronchial epithelium and the umbilical cord as well as blood and serum. Other tissues have similar miR-21-5p levels without displaying the increased NTA-U, so this pattern cannot be explained by an expression bias of miR-21-5p. Nevertheless, the small fraction of miR-21-5p reads taken by this isoform, <8%, suggests moderate relative abundance only. Recent work on miRNA kinetics has proposed that miRNA transcripts become uridylated after mRNA targeting (32). Unfortunately, this NTA#U isoform is not tagged as validated by previous data because the uridylation decrease detected after TUT4/7 knockdown was not significant for miR-21-5p (27). This kind of observation is prototypic for the usefulness of isomiRdb in generating new hypothesis and in validating the expression background at miRNA and isomiR level.

### Use case: a 5′ length variant of miR-181a-5p is almost exclusively detected in urine

Besides their function as key regulatory elements of gene expression, miRNAs hold great interest as biomarkers among other properties because of their stability in bodily fluids. Several studies partially compiled elsewhere (28) have attempted to define circulating miRNA disease biomarkers. In this context, urine biomarkers could be particularly non-invasive, an important aspect for continuous monitoring. One possible example of an interesting biomarker candidate is miR-181a-5p, a miRNA that different studies have reported to have predictive value of therapy outcome (33,34). Using the *miRNAs* view of isomiRdb, we can quickly check that miR-181a-5p is highly expressed in a wide range of tissues and particularly in urine. Furthermore, we can also obtain the isomiR profile using the *Heatmaps* page. The expression heatmap reveals that even though miR-181a-5p is already quite urine specific (0.83 TSI) there is a 5′ length variant isoform, *ACATTCAACGCTGTCGGTGAGT*, that is almost exclusively present in this fluid (20 413 RPM versus <50 RPM elsewhere). Furthermore, by inspecting the fraction of miR-181a-5p reads assigned to this isoform, the pattern appears even more striking: the relative abundance in most other tissues comes close to 0% compared to 88% in urine. Using isomiRdb downloadable metadata, we confirmed that urine samples (*n* = 63) came from four different studies, which made us discard the influence of any protocol specific artifact. Moreover, the quality control pipeline tagged this variant as likely *bona fide* because alternative Drosha processing for miR-181a-5p had previously been reported (26). Nonetheless, such prominent differences should be taken with caution. This example illustrates a basic data exploration using isomiRdb and how users can benefit from the hosted quality tags and sample information to increase support of their analysis.

## DISCUSSION

IsomiRs are widespread and expressed on a cell-type dependent manner. Different studies have proposed physiological roles for miRNA variants. For instance, miRNA transcripts seem to get monouridylated because of target regulation, a modification that destabilizes them (32). More recently, 3′ uridylation has been linked to alternative target repression in specific miRNAs (35). Another recent study describes that 1-nt-short isomiRs typically accumulate in several cancer tissues and cancer cell lines (36). Interestingly, the same work explains how RDR1, an exogenous plant immune protein, can add mononucleotides to the 3′ end of these isomiRs to restore the length of their canonical sequence in mammalian cells. Moreover, these modifications resulted in antitumor effects in *in vivo* mouse models. Not surprisingly, several studies consider isomiRs in the quest for disease biomarkers (10,37). Remarkably, some models successfully distinguish between different types of cancer. Although sequencing errors may seemingly inflate the number of isoforms in a sample, there is currently plenty of evidence to support a role of isomiRs beyond mere experimental artifacts. We have taken advantage of previously available evidence on Drosha processing (26) and TUT4/7 monouridylation patterns (27) to curate biologically relevant isomiRs, a feature that radically increases confidence on entries hosted in isomiRdb.

Despite their increasingly apparent importance, until now few resources are available to query detected isomiR sequences as well as their abundance. The two prominent examples are IsomiR Bank (19), with a thoroughly curated dataset, and TIE (20), based on TCGA data. IsomiR Bank that excels by its multi-species support, relies on a relatively low number of samples which complicates tracking of less common miRNA variants. On the contrary, TIE builds on >10 000 samples and has a clear scope to one organism (human) and a disease focus (cancer). Although most isoforms will still be present in cancerous tissue, their expression levels are probably altered by the disease. Besides, TCGA does not cover all human tissues and cell types, making it an insufficient resource to be used as background control for isomiR-based biomarker discovery. IsomiRdb aims to provide a comprehensive view on high- and low-abundance isomiRs. It offers an exceptional range of samples, tissues and cell types with plenty of coverage to detect even the lowliest expressed isoforms. Of course, such a comprehensive compilation of isomiRs unavoidably contains noisy and false positive isoforms (artifacts originated by library construction or sequencing), particularly those that are only present in a handful of experiments. To account for this issue, we performed quality control on all isomiR entries to flag potential artifacts and certify likely biological isoforms. Furthermore, we base the main part of the resource on higher abundance isomiRs by setting a threshold of 1 RPM in 1% of the samples. Although any threshold of this nature can appear arbitrary (we keep approximately the top 3% isoforms), we understand it's necessary to relegate isomiRs that are not widespread enough in the interest of clarity. Nevertheless, users can still explore if any isoform has previously been detected at all and in how many samples, which can be useful in some situations like for isomiRs only expressed in tissues underrepresented in isomiRdb. With 3 million detected isomiRs across 42 499 miRNA-seq samples, isomiRdb comes close to the limit of naturally occurring isoforms in known miRNAs (an average of ∼1200 isomiRs per mature miRNA) and it's unlikely that many true isomiRs have escaped our analysis. The limitations inherent to the annotation or the sequencing process might be an exception in this regard.

Besides the obvious interest of isomiRdb as an isomiR expression database, the very broad collection of miRNA-seq expression data, in terms of the number of samples and sequencing reads, is remarkable. The compiled dataset can be used to answer several research questions related to the expression of miRNAs. One current limitation of isomiRdb is that it does not provide validation information on isomiR processing by Dicer or TENT2, two enzymes that have long been established to participate in miRNA metabolism and that can generate alternative miRNA transcripts. Future releases of isomiRdb should include quality tags supporting or refuting involvement of these enzymes in a similar way to the already present tags for TUT4/7 and Drosha. Another important limitation of isomiRdb compared to similar resources (19) is that it is focused on human samples. Although other species are not nearly as present in SRA or other publicly available sources, it would be of much interest to include comparably annotated datasets from evolutionarily related species to explore conserved patterns in the expression of miRNAs and isomiRs. Such analysis would not be without challenges given the dissimilar quality of miRNA annotations across species, although they could be mitigated by use of an evolution aware database like MirGeneDB (38). In this context, *Mus musculus* is probably the most appropriate candidate model organism to be included in future releases of isomiRdb given its interest to the research community, the quality of its annotation and the abundance of miRNA-seq data available on SRA, only behind *Homo sapiens*. We thus initiated to annotate and process *Mus musculus* miRNA-seq experiments in the same manner that we originally did for human samples with the goal of incorporating them to isomiRdb in the foreseeable future.

## DATA AVAILABILITY

isomiRdb website is available at: https://www.ccb.uni-saarland.de/isomirdb. All data stored in this database is available in the Downloads section.
